# Essay on Pivot Teeth

**Published:** 1848-10

**Authors:** Charles Bonsall


### AN ESSAY ON PIVOT TEETH.

***Mr. President, and Gentlemen of the Society:***

At our last annual meeting, I was requested to prepare an address on the subject of "Pivot Teeth;" I frequently thought of sitting down to it, when I have had a few hours of leisure, but then again, upon consideration, I despaired of being able to advance anything that was not known to every member of the Society who might be present, and so, from time to time, have postponed the subject, until now, when only a few hours preceeding the meeting, when being much engaged, I am reminded that it must be done, so I will try and give you whatever comes to my recollection, in the short time I can spare.

The practice of engrafting a substitute for decayed or broken teeth, upon the remaining stump, has been in vogue from the earliest times of dental practice, though at first very rudely performed, the substitute being made of the teeth of men or some one of the lower order of animals, liable to frequent decay and discoloration, and of course requiring frequent renewal.

Since the art of making and coloring porcelain teeth has been brought to the state of perfection it has now arrived at, there are much stronger inducements to use false teeth, and particularly pivot teeth, than formerly, as when once properly set, they may always be expected to last so long as the root continues sufficiently solid and firm to retain them, with only an occasional new pivot, say every two or three years. My own practice as to inserting pivot teeth is, to only recommend them when all the teeth required can be inserted in that manner, and this supposes that they are all either incisors or canine teeth, as the roots of the bicuspids or molars, are rarely of a form to permit lasting pivot teeth to be inserted on them; for myself, I hardly ever attempt it, if any of the teeth require to be set on plate, then I think it preferable to fix them all in that manner, as being more permanent, and the comfort and usefulness of plate teeth is increased to a great extent in proportion to the increased number of them—in cases where one or more of the front teeth are too much decayed to be preserved by plugging, and the rest of the arch is complete, and the teeth sound, I particularly recommend pivot teeth, to obviate the necessity of filing between sound teeth, molar or bicuspids, for the purpose of affixing clasps. When we have decided to insert a pivot tooth, I first begin by sawing the tooth *in* a considerable depth on front and either side where I can approach it, as close up to the gum as convenient, then, with a pair of excising forceps cut off the crown; then take a fine drill shaped on purpose, or watchmaker's brooch, firmly set into a handle, and not too long, a portion of the brooch extending beyond the handle for fear of breaking it off; I now caution the patient in case I suspect any vitality in the nerve, to avoid any sudden movement, informing them that I shall probably cause severe pain for an instant, then poise the instrument carefully over the nerve in the proper direction, to be carried by a sudden pressure entirely to the end of the root, when with a half turn on withdrawing the instrument, the nerve is usually found entirely destroyed, and we are enabled to increase the size of the dental canal, so as to fit it for a pivot without any pain to the patient. During the progress of drilling, we should withdraw the instrument two or three times to examine whether we are keeping in the centre of the root—as to depth, I am governed by the probable length of the root. When the hole is deep enough I

take either an oval file of suitable size, or a half round gunsmith's file, of say four or five inches long, and file down the stump rather below the gum. I now select a tooth matching in shape and color as near as possible to the adjoining teeth, and take care to fit it well to the root inside and outside, then taking a piece of pivot wood, which should be either of tough, well-seasoned hickory, or what is in Pennsylvania called arrow or skewer wood, I cut a shoulder of the proper length, and then dress it down with a file until it can just be forced into the tooth by a gentle pressure; this being done I next measure the depth of the hole and cut off the pivot of the proper length, and file it down until I find it will just enter the opening, we now very carefully dry the hole in the root by means of a little cotton on the end of a small drill, then taking a towel to prevent hurting the finger, press it up till it rests solidly on the root, and it is finished.

In putting in a pivot for the first time it is much better to use one merely thick enough to retain it in its place, without dropping out, and in a few days replace it by a thicker one; as in this way the patient is saved much pain and swelling of the face, which formerly, was considered an almost necessary consequence of having pivot teeth inserted. In cases where there is much appearance of inflammation at the root, and consequent looseness, I think it best to decline inserting a tooth on pivot, as it will seldom give satisfaction to the patient, and the reputation of the operator, as well as that of the dental art generally, is injured in the estimation of the patient, and through him, others of the community.

While on this subject, let me express the great regret I frequently feel at finding operations of different kinds which have been performed on the teeth, by respectable dentists, which they must have known were contrary to correct dental principles, and could only have been performed to satisfy a request of the patient, who could not know they were improper, *or else* for the sake of the pay to be received, either of which are very inadequate reasons, as I hold that a patient should never employ a dentist, unless with full confidence in his knowledge and ability, and then the dentist should be governed solely by his judgment of what is the most correct practice in the case. If we would all follow this rule strictly, it would not only increase our own reputation and business, but also elevate the science of dental surgery in the estimation of the community, to the high stand which it deserves to attain. I must acknowledge however, and I do it with pleasure, that I notice from year to year, a higher stand taken by my professional brethren, and devoutly hope it may continue and increase.

I had thought when I commenced, to go into something of a history of the various so-called improvements which have been proposed at different times, some of them quite complicated, and looking very well in theory, but not near so well in practice as the simple method which I have described, aud which is I believe, almost universally used at present. In closing I cannot but regret that I have not been able to give you anything more interesting than this essay, but my engagements will not permit more at present. CHARLES BONSALL.

Sept. 5th, 1848.

